# Enhanced Olivine Reactivity in Wet Supercritical CO_2_ for Engineered Mineral Carbon Sequestration

**DOI:** 10.1021/acs.energyfuels.4c04120

**Published:** 2024-10-28

**Authors:** Mohamed A. Saleh, Huw Shiel, Mary P. Ryan, J. P. Martin Trusler, Samuel Krevor

**Affiliations:** † Department of Earth Science and Engineering, Imperial College London, Exhibition Road, London SW7 2AZ, U.K.; ‡ Department of Chemical Engineering, Imperial College London, Exhibition Road, London SW7 2AZ, U.K.; § Department of Materials Science, Imperial College London, Exhibition Road, London SW7 2AZ, U.K.

## Abstract

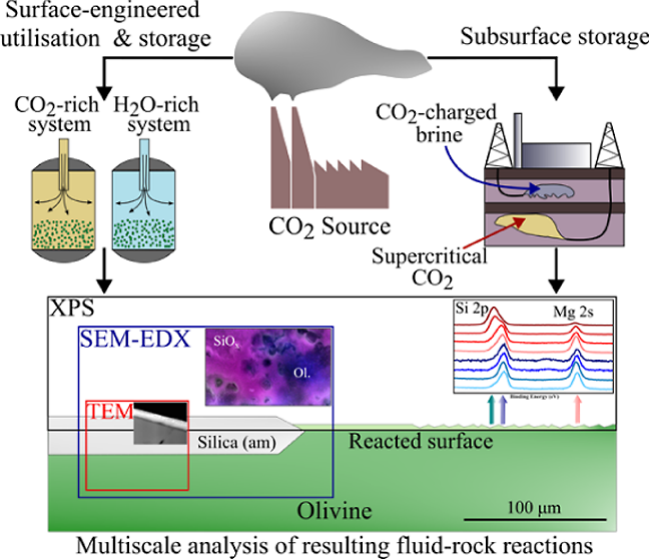

The success of CO_2_ mineralization as a potential solution for reducing carbon emissions hinges on understanding chemical interactions between basaltic minerals and CO_2_-charged fluids. This study provides a detailed analysis of olivine dissolution in CO_2_-water mixtures at 90 and 150 °C, 2–9 MPa, and for 8 and 24 h, in both water- and CO_2_-dominant conditions. By using olivine crystal sections instead of powders, surface agitation is prevented, closing the gap between laboratory studies and natural settings. Surface chemistry, texture, and cross-sectional properties were examined pre- and postreaction using a multiscale approach combining spectroscopic and imaging techniques. Results show that wet supercritical CO_2_ environments lead to significant olivine dissolution, forming Mg-depleted, Si-enriched etched surfaces, and under certain conditions, the formation of passivating silica precipitates. In contrast, reactions in aqueous fluids caused minimal changes in surface chemistry and texture with no silica precipitation. These observations indicate that reaction extent in the CO_2_-rich phase is greater relative to water-rich mixtures at equivalent temperature, pressure, and reaction duration. The presence of silica precipitates incorporating leached metals indicates limited transport of reactant away from reaction sites in a CO_2_-rich medium. This study semi-quantitatively evaluates reaction extents in both CO_2_-rich and aqueous systems across a wide range of parameters, demonstrating faster mineralization in CO_2_-rich environments and highlighting their potential for enhancing the CO_2_ storage efficiency.

## 1 Introduction

Engineered carbon dioxide mineralization is an emerging sequestration technology that uses fluid–rock reactions to ensure the permanent trapping of CO_2_ in a thermodynamically stable solid mineral form. Mineral trapping boasts a vast storage potential and offers a means to mitigate challenges presently associated with geological carbon storage in sedimentary reservoirs, such as leakage and reliance on costly seismic monitoring techniques.^1,2^ Field-scale CO_2_ mineralization operations in CarbFix at Hellisheidi in Iceland and the Wallula Project in the USA have demonstrated the potential of engineered mineral trapping for geological CO_2_ sequestration.^3,4^ The fractured basalts rich in forsteritic olivine constituting the reservoirs at Hellisheidi (∼15 wt % olivine) and Wallula (∼10 wt % olivine) are regarded as the optimal rocks for CO_2_ mineralization for reasons of reactivity with CO_2_ and high divalent cation content (primarily magnesium and calcium) required for carbonate formation.^5−8^ In addition to geological storage, surface-engineered olivine mineralization processes provide an alternative means of sequestering carbon, with the potential benefit of obtaining valuable metal byproduct.^9,10^

Most olivine mineralization research has focused on reactions taking place in water-rich reaction systems. The formation of passivating reaction products and the kinetics of olivine dissolution reactions have been extensively studied in the presence of CO_2_-charged solutions using batch reactor experimental setups.^11−16^ These studies provide evidence of an initially nonstoichiometric dissolution mechanism that contributes to the formation of a Si-rich leached layer and dense silica layers which rapidly mechanically passivate the reactive olivine surface. Water-bearing supercritical CO_2_ (scCO_2_) offers an alternative reaction environment commonly present at subsurface reservoir conditions.^17^ In situ spectroscopic analysis of olivine carbonation in wet supercritical CO_2_ revealed anhydrous carbonate formation at lower temperatures compared to water-rich systems, indicating a highly reactive environment.^18^ An important factor for reactivity in scCO_2_ is that the fluid phase is at or beyond saturation with respect to water, allowing for the formation of thin water films on the mineral surface where the reactions can take place.^8,19,20^

Key aspects of CO_2_ mineralization in olivine remain inadequately understood. Existing studies assessing product layers on olivine powders are prone to bias due to the mechanical removal of surface products caused by particle abrasion during stirring.^21^ This phenomenon influences the morphology, thickness, and mechanical properties of the layers and prevents reliable assessments of surface passivation. This is compounded by a lack of quantitative assessment of the dissolution extent of olivine in wet scCO_2_ compared to aqueous solvents. Direct comparisons of the extent of dissolution reaction across water-rich and CO_2_-rich environments are required to validate the claims of enhanced reactivity in the scCO_2_ system for which we do not have a kinetic rate model. Previous comparisons of reactions in aqueous (CO_2_-charged) solutions and scCO_2_ do not evaluate the influence of temperature, pressure, and reaction time on the reaction layer.

In the present study, we utilize the formation of silica-rich phases on the mineral surface of forsteritic olivine as a semiquantitative indicator of reactivity comparing reaction in water-rich and CO_2_-rich fluid phases. The use of pure crystal planar surfaces allows the investigation of interfacial regions without complexities arising from agitation during the experiment, providing a distinct advantage over powder-based experiments. The aims of this study are three-folds: to investigate olivine dissolution in CO_2_-water systems (wet scCO_2_ and aqueous) under varying reaction conditions to identify differences in reaction progress; to analyze surface chemistry and textures that may inhibit reaction progress using a multiscale approach; and to assess the implications of these reactions for optimizing the efficiency of engineered mineral carbon sequestration. To address these points, a series of batch reactor dissolution experiments were completed at a range of temperature and pressure conditions. Conditions emulate both subsurface storage settings where both fluid systems can exist and surface-engineered settings where scCO_2_, if more reactive, could allow for more favorable operating conditions.

## 2 Experimental Section

### 2.1 Materials

**Figure 1:**
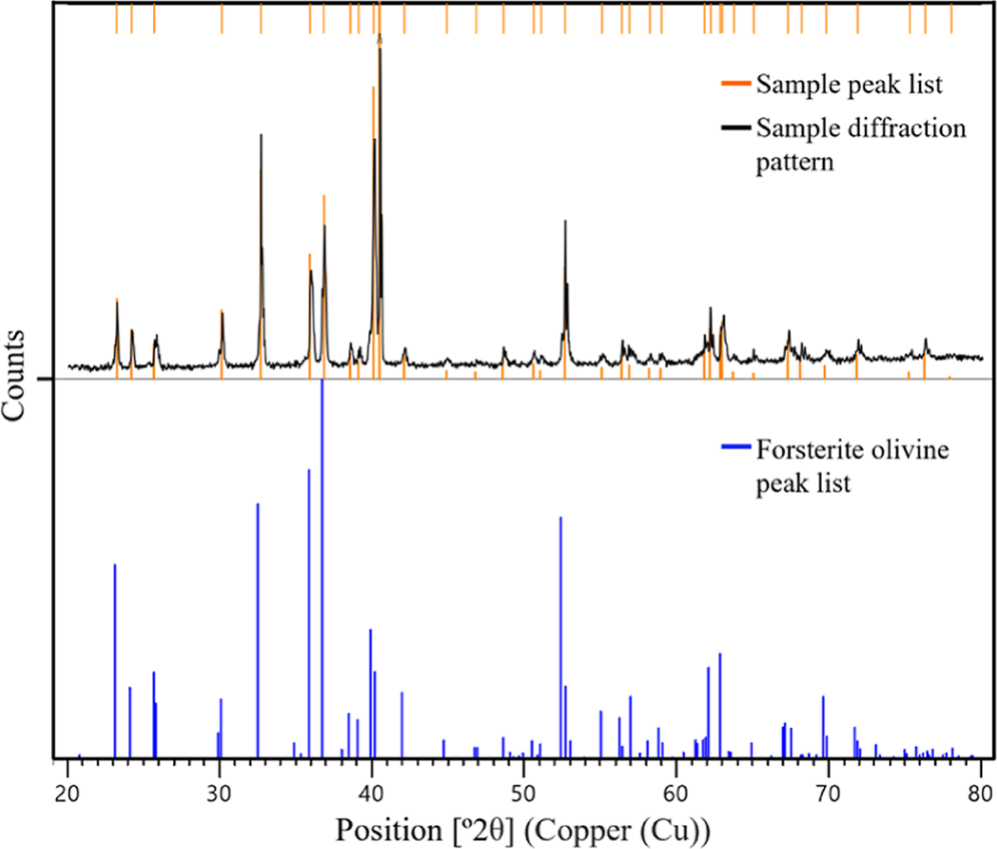
XRPD pattern for the crushed olivine crystals (black) were collected using a Kα1 copper source, with a step size of 0.0340° (2θ) and a scan step time of 208.0 s. The identified sample peaks (orange) were best matched to forsterite olivine Mg_1.72_Fe_0.28_SiO_4_ (blue), based on the International Centre for Diffraction Data library (no. 01-079-1200).

Magnesium-rich olivine with a stoichiometry of Mg_1.72_Fe_0.28_SiO_4_ was procured from Sibelco Nordic AS, Åheim, Norway (Table 1) (Figure 1). Centimeter-scale crystals were cleaved to produce a total of 16 rectangular ∼10 × 10 × 4 mm sections using a Buehler IsoMet water-cooled cutter wielding a high-concentration diamond blade (see Figure S1). To avoid contamination of the planar surfaces, the cut surfaces were not chemically treated. Polishing was deliberately avoided as it mechanically alters the crystal structure, introducing additional broken bonds that could compromise surface integrity and affect dissolution behavior.^15^ All samples were subsequently washed in an aqueous isopropanol solution, oven-dried at 60 °C, and weighed before each test.

**Table 1:** Sibelco Olivine Chemical Composition (LOI)

chemical compound		% by weight
MgO	magnesium oxide	49.0
SiO_2_^a^	silicon oxide^a^	41.3
Fe_2_O_3_	iron oxide	7.4
Cr_2_O_3_	chromium oxide	0.35
Al_2_O_3_	aluminum oxide	0.49
CaO	calcium oxide	0.19
NiO	nickel oxide	0.31
MnO	manganese oxide	0.09
Na_2_O	sodium oxide	0.03
K_2_O	potassium oxide	0.04
L.O.I^b^	loss on ignition^b^	1.9
moisture		0.8

a Present as silicate.

b wt % after 30 min at 900 °C/air.

Prior to the experiments, thick-sections were analyzed using scanning electron microscopy (SEM) with energy-dispersive X-ray spectroscopy (EDXS). The absolute atomic percentage of magnesium naturally varied from 26 to 30 at %. No surface impurities were detected with the exception of minor occurrences of Ca-rich crystallites consistent with diopside (Ca_*x*_Mg_*y*_Al_*z*_Si_2_O_6_) minerals commonly found in association with olivine (see Figure S2).

### 2.2 Stirred Batch Experiments

A total of 16 batch experiments were conducted under conditions of controlled temperature and pressure and maintaining CO_2_ and aqueous fluids saturated with respect to each other (reagents in Table 2). Eight experiments investigated the reaction of olivine and CO_2_ in an aqueous solution (0.2 M NaCl). The remaining reactions were conducted in wet scCO_2_. Samples were reacted in each reaction system for periods of 8- and 24 h, with two repeats carried out for each condition.

**Table 2:** Details of Reagents Utilized in This Study

compound	source	purity
carbon dioxide N4.5 (CP grade)	BOC	99.995%
sodium chloride ACS reagent	Sigma-Aldrich	≥99.0%
purified water	Merck Millipore Direct-Q 3UV	DDI (18.2 MΩ cm)

**Figure 2:**
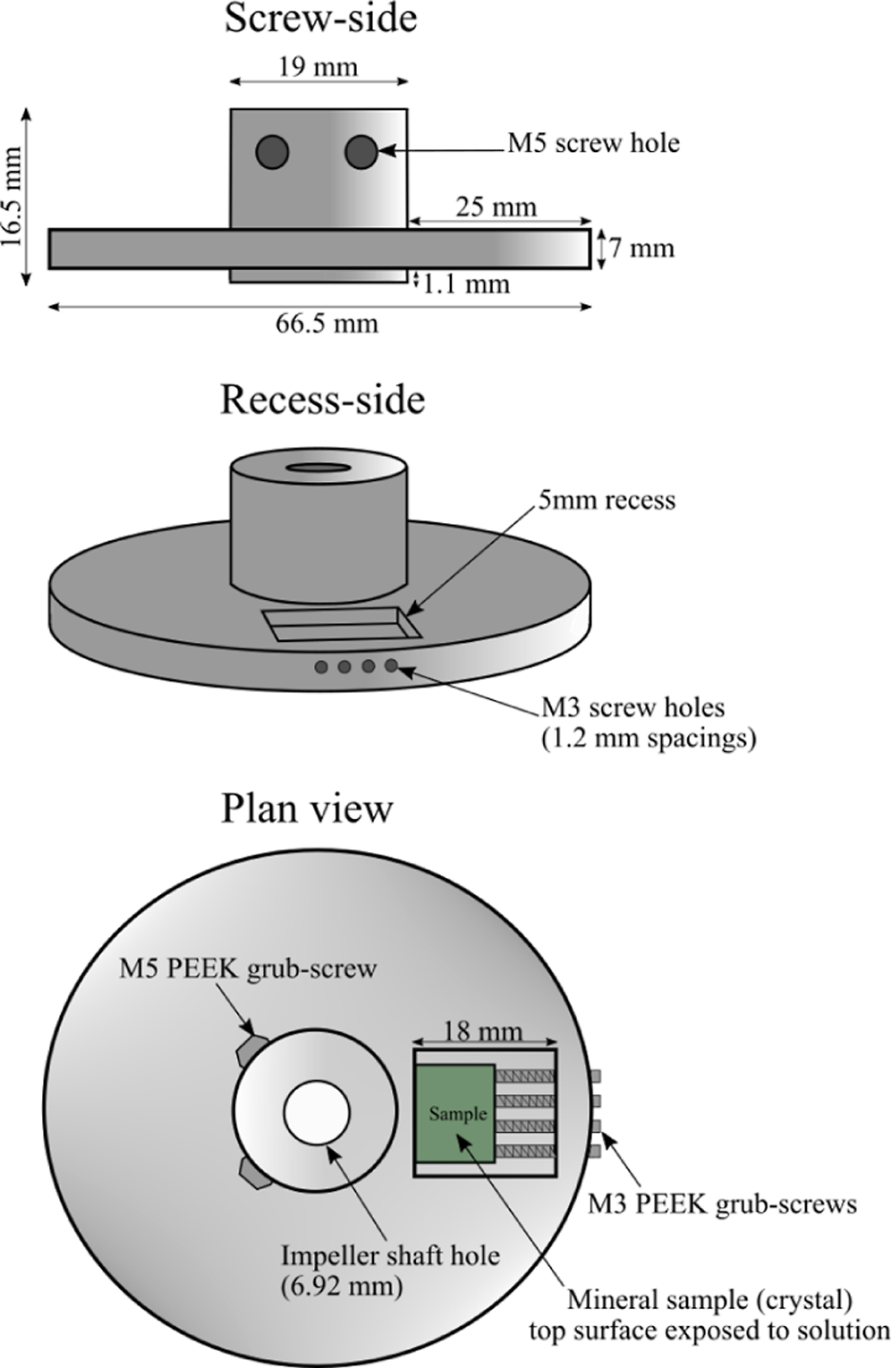
Schematic diagrams of the PEEK sample holder designed for crystalline olivine thick-section experiments. This was held at the bottom end of the stirring rod (see Figure 3).

In each batch, a single olivine thick section was placed inside a High-Pressure Series 1200 mL Hastelloy reactor vessel (model 4540, Parr Instrument Company). Temperature was monitored via a Type J thermocouple housed in the head thermowell and connected to a temperature controller. A sample holder was machined from polyetheretherketone (PEEK) high-resistance polymer and attached to the stirring shaft to secure samples in place during rotation while eliminating the need for epoxy resins or steel components which may degrade and contaminate the system overtime (Figure 2). Each sample is fixed within the recess by using PEEK screws (Figure 2). The setup includes a set of four screw ports, providing flexibility to accommodate different sample geometries as needed.

**Figure 3:**
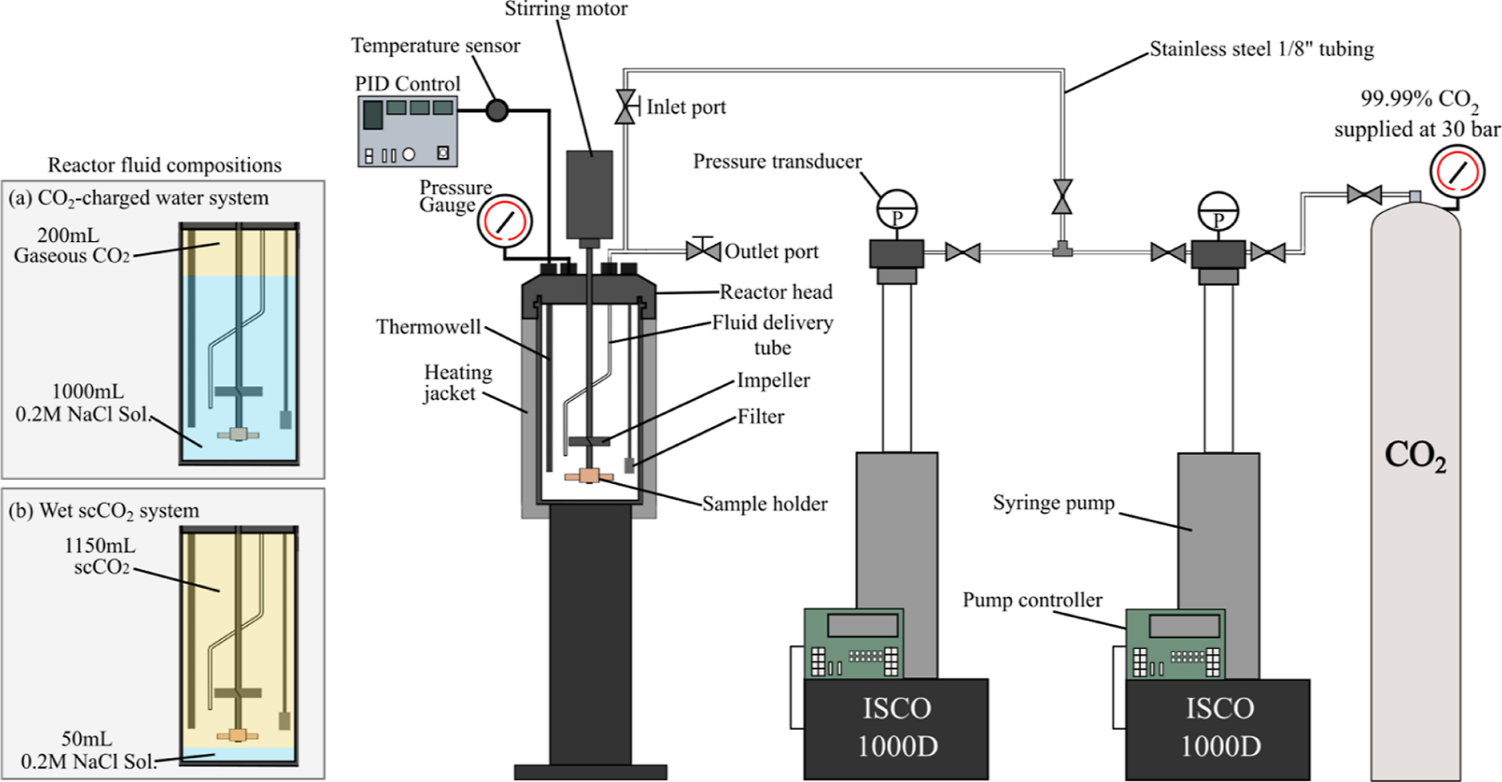
Process schematic of the batch reactor setup for olivine dissolution reactions showing the reactor compositions in (a) water-rich and (b) CO_2_-rich (wet scCO_2_) systems.

The first set of experiments in water-rich mixtures was conducted at 90 °C (±2 °C) and 2 MPa to match the upper limit of reservoir temperatures and injection pressures at CarbFix^22,23^ (see Table 3). The second set of experiments prioritized enhanced dissolution kinetics at 150 °C (±2 °C) and 6 MPa. Each sample was submerged in 1000 mL of 0.2 M NaCl solution (Figure 3). Once the target temperature was reached, two cycles (400 mL) of 99.99% purity CO_2_ gas supplied at 3 MPa were injected to purge air from inside the vessel. A ThermoFisher Scientific GLD Pro gas leak detector was placed at the release valve outlet to confirm that CO_2_ had displaced the air inside the reactor. A 200 mL headspace of CO_2_ was maintained by a syringe pump (1000D, Teledyne Isco). Changes in the CO_2_ pump volume (mL) were monitored at constant temperature and pressure using pump controls and allowed to stabilize. Equilibration time was calculated at 4 h for water-rich mixtures and included as part of the total reaction time. Stirring was set at 100 rpm ensuring that mass transfer to the mineral surface is not a limitation on the rate of reaction. Reactions were terminated by emptying the vessel through the outlet valve. Samples were then unloaded and placed in a vacuum oven at 60 °C overnight before analysis.

**Table 3:** Summary of Batch Reactor Experiment Conditions and Durations

sample code	CO_2_ condition	reaction time (h)	carrier solution	*P* (MPa)	*T* (°C)	stirring rate (rpm)	vol. CO_2_ (cm^3^)	vol. solution (cm^3^)	capacity for scCO_2_ saturation w.r.t H_2_O^b^
Subsurface Reaction Conditions									
1A (X^a^)	aqueous sol	24	0.2 M NaCl	2	90	100	200	1000	
1B (Z^a^)	wet scCO_2_	24	0.2 M NaCl	9	90	100	1150	50	250%
1C (J^a^)	aqueous sol	8	0.2 M NaCl	2	90	100	200	1000	
1D (K^a^)	wet scCO_2_	8	0.2 M NaCl	9	90	100	1150	50	250%
Enhanced Reaction Conditions									
2A (X^a^)	aqueous sol	24	0.2 M NaCl	6	150	100	200	1000	
2B (Z^a^)	wet scCO_2_	24	0.2 M NaCl	9	150	100	1150	50	250%
2C (J^a^)	aqueous sol	8	0.2 M NaCl	6	150	100	200	1000	
2D (K^a^)	wet scCO_2_	8	0.2 M NaCl	9	150	100	1150	50	250%

a Denotes a repeat run and its associated sample code.

b Denotes the water available within the scCO_2_ system, estimated to be sufficient to exceed the saturation capacity of the scCO_2_ volume by 250%.

The third and fourth set of reactions were conducted in the wet scCO_2_ system at 90 °C (±2 °C), 9 MPa and 150 °C (±2 °C), 9 MPa conditions, respectively. The volume of water required to fully saturate 1200 mL of scCO_2_ was calculated to be ∼20 mL using water solubility in CO_2_ data from models in ref (24). To ensure water is not the limiting reactant, 50 mL of 0.2 M NaCl solution was placed in the reactor, allowing any water solvated in the CO_2_-rich phase to be buffered by liquid water. The sample holder was positioned 2.1 cm above the water level (Figure 3) to ensure that the sample was entirely suspended within the CO_2_-rich phase and prevent the reactive mineral surface from submerging in solution. Once internal temperature reached the desired point, air inside the vessel was purged with 2000 mL of CO_2_ gas_._ System pressure was incrementally ramped up using the twin pump setup (Figure 3) until desired pressure was reached. Aqueous experiment run times were set as benchmarks for scCO_2_ investigations. A stirring rate of 100 rpm was applied to maintain consistency with water-rich tests, and to limit condensate accumulation on the sample holder during depressurization at the end of reaction time.

### 2.3 Kinetic Modeling

Kinetic models of the water-rich systems were developed using PhreeQC v3.7.3 software to numerically simulate the rate of Mg and Si release, solution pH calculations, and mineral saturation indices at conditions utilized in this study. The Lawrence Livermore National Laboratory thermodynamic database (llnl.dat) was utilized to run the simulations. Pressure of the CO_2_ headspace was maintained constant throughout the batch simulations. The rate expression was applied to describe the dissolution of forsterite from 90 to 150 °C.^25^ In acidic solutions, the dependence of dissolution on pH is described as a power law, whereas the dependence on temperature is exponential (eq 1)
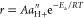
1Here, *r* (mol cm^–2^ s^–1^) is the dissolution rate, *A* = 0.0854 mol cm^–2^ s^–1^ is an empirical pre-exponential factor, *a*_H+_^*n*^ accounts for the influence of hydrogen ion activity with *n* = 0.45 and 0.48 being the reaction order for forsterite dissolution at 90 and 150 °C, respectively, and *E*_a_ = 60.2 and 43.9 kJ mol^–1^ is the activation energy at 90 and 150 °C, respectively. Average olivine thick section dimensions (10 × 10 × 4 mm) were used to calculate a sample surface area representative of our sample set and incorporated into the model.

### 2.4 Analytical Methods

**Figure 4:**
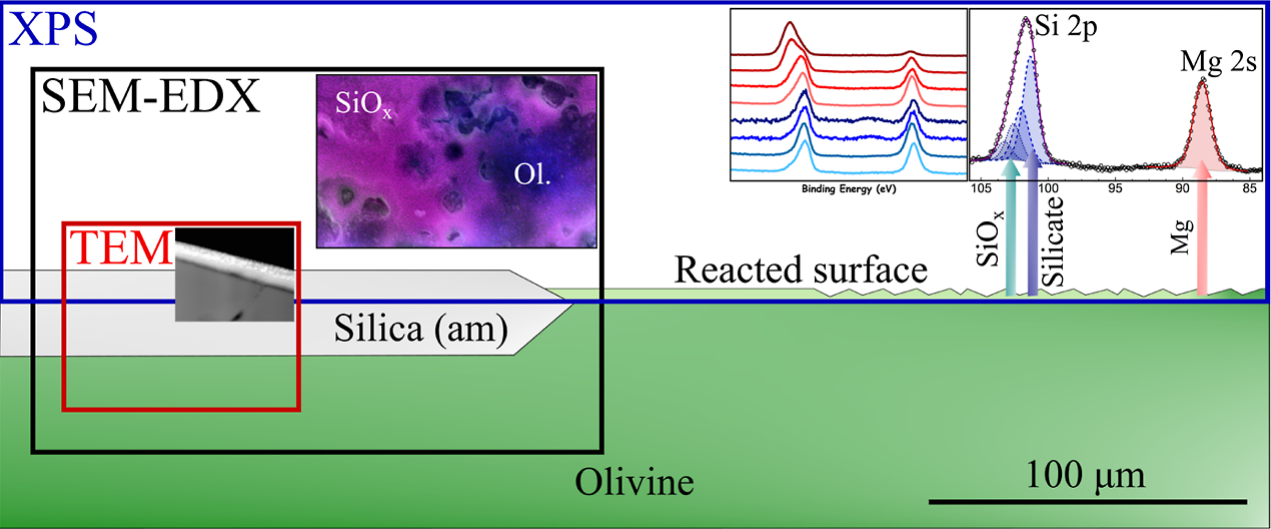
Multiscale hierarchical surface analysis techniques employed to examine the planar surface of forsteritic olivine reacted in water-rich and CO_2_-rich environments.

To investigate the reactive surface properties in detail, our study utilizes a comprehensive, multiscale analysis approach involving techniques such as SEM-EDXS, X-ray photoemission spectroscopy (XPS), and transmission electron microscopy (TEM) (Figure 4).

Surface chemical composition and evolution were assessed using SEM-EDXS and XPS. Microstructural characterization was completed using an FEG SEM (Quanta FEI 650). Samples were coated with a 14.9 nm chromium coat to minimize charging. EDXS data were acquired at a nominal depth of 1 μm via Bruker Nano ESPRIT spectrum and mapping tool using a XFlash 6|60 detector with 70° tilt angle. EDXS was utilized to generate elemental composition maps and spectra of the surface.

XPS data were acquired using a Thermo Scientific K-Alpha Surface Analysis instrument. Monochromated Al kα X-rays (1486.6 eV) were incident on the sample with a spot size of 400 μm, and an automated flood gun was used to prevent sample charging. Survey scans were acquired using a pass energy of 200 eV and a step size of 0.5 eV. Detailed core level spectra were acquired at a pass energy of 20 eV and a step size of 0.1 eV. Core level spectra were fitted using Thermo Scientific Avantage software, utilizing smart Shirley background subtraction and Voigt line shapes.

Cross-section analysis of nanometer-micrometer scale features was conducted using TEM (JEOL JEM-2100F) (Figure 4). Sample 2B (24 h, wet scCO_2_) was chosen for TEM and scanning TEM (STEM)-EDXS compositional analysis based on the extensive product layers formed on its surface. Lamella were extracted from the selected samples using focused-ion beam (FIB) milling completed using gallium in a ThermoFisher HELIOS 5 CX instrument. A protective layer of platinum was applied to the target area prior to milling. A section measuring ∼10 μm × 9 μm was produced from each sample and placed onto a copper grid prior to analysis.

## 3 Results

### 3.1 Models of Aqueous Experiments

**Figure 5:**
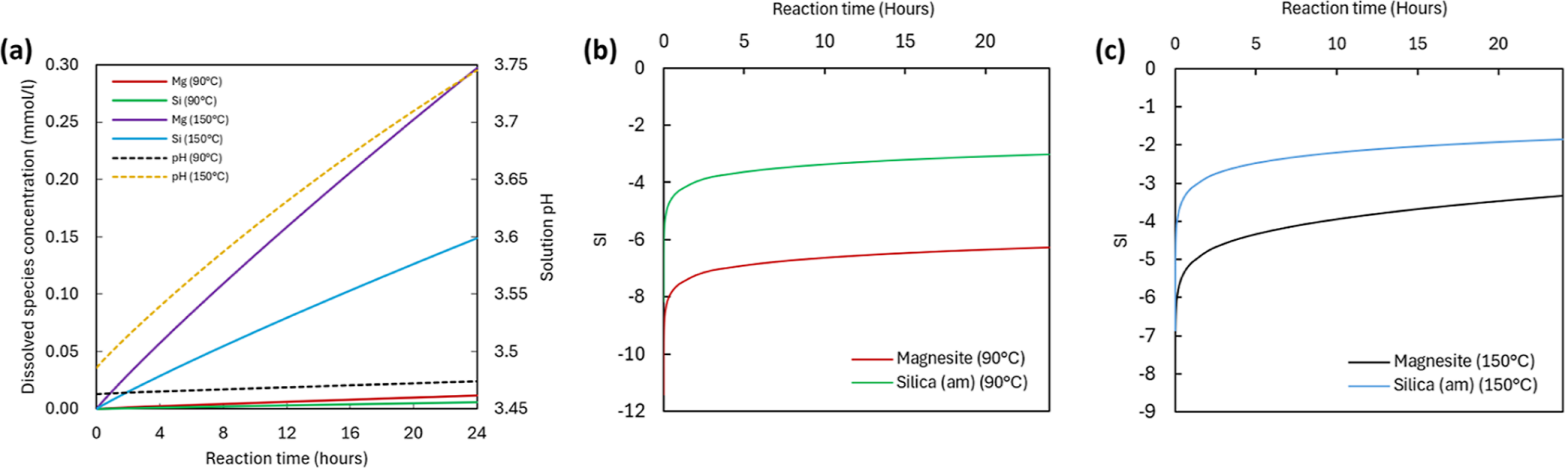
(a) Predicted dissolved Mg and Si concentrations and calculated pH profiles after reaction in 90 °C, 2 MPa and 150 °C, 6 MPa water-rich (0.2 M NaCl aqueous solution) experiments. (b,c) Saturation indices of magnesite (MgCO_3_) and amorphous silica (SiO_2am_) plotted against reaction time for (b) 90 and (c) 150 °C experiments. Neither magnesite nor silica is supersaturated within the given reaction timeframes.

Kinetic models of aqueous solution experiments conducted at 90 °C, 2 MPa and 150 °C, 6 MPa conditions are shown in Figure 5. Figure 5a compares the rate of Mg and Si release in both temperatures and the pH evolution of the aqueous solution within the 24 h reaction window investigated in this study. In both temperature settings, geochemical models indicate that precipitation of the passivating amorphous silica phase is not achieved in aqueous solutions during the reaction time frame (Figure 5b,c). Similarly, the simulations do not predict the formation of any magnesium carbonate phase.

### 3.2 Surface-Localized Chemical Analysis

**Figure 6:**
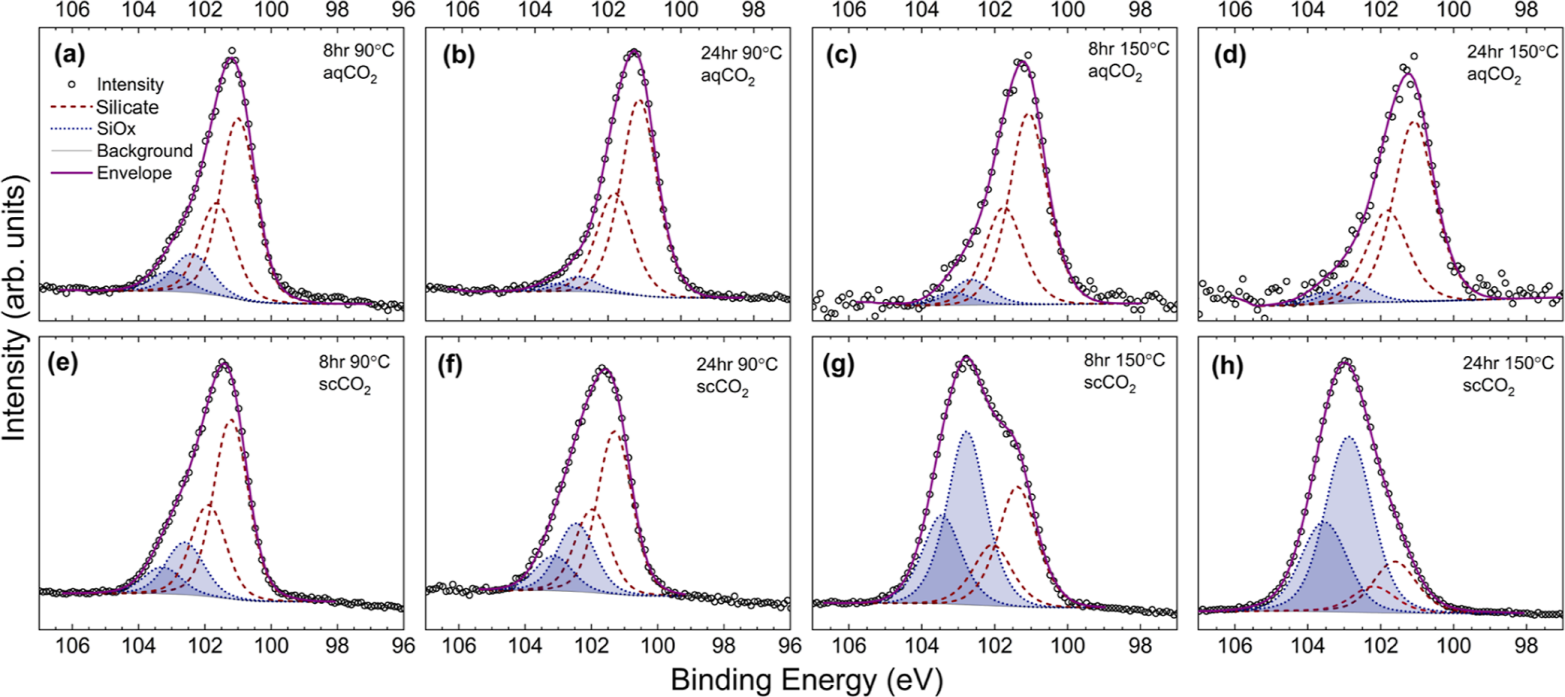
XPS Si 2p core level spectra of reacted surfaces in (a–d) water-rich and (e–h) CO_2_-rich reaction systems for different durations and temperature conditions. Each peak consists of two doublets; two peaks (red, dashed line) attributed to the forsteritic olivine silicate phase (labeled silicate), and two peaks (shaded blue, dotted outline) attributed to a silica layer formed during reaction (labeled SiO_*x*_).

Surface chemical composition was studied using XPS with an aerial spatial coverage of 0.13 mm^2^ and sensitivity within 10 nm from the surface, revealing changes in the bonding environment and oxidation state after reactions. Examination of the Si 2p core level (Figure 6) reveals a primary peak at 101.5 eV, with a clear shoulder to higher binding energy that indicates a second chemical state at approximately 102.5 eV. Deconvolution of the Si 2p region through peak fitting confirms the presence of two chemical states. The dominant contribution at lower binding energy is attributed to the forsterite olivine (silicate) phase, as determined from unreacted samples (Figure S4a) and consistent with previous reports.^26^ The second doublet is consistent with the binding energy of SiO_*x*_ (*x* ≤ 2).^27^ Si 2p core level fitting of an unreacted surface does not require and will not accommodate this second doublet, indicating that the SiO_*x*_ detected exclusively in reacted samples is formed during the batch reactor experiments. Detailed Si 2p core level spectra from the water-rich and CO_2_-rich reaction systems are shown in Figure 6a–h, respectively.

**Figure 7:**
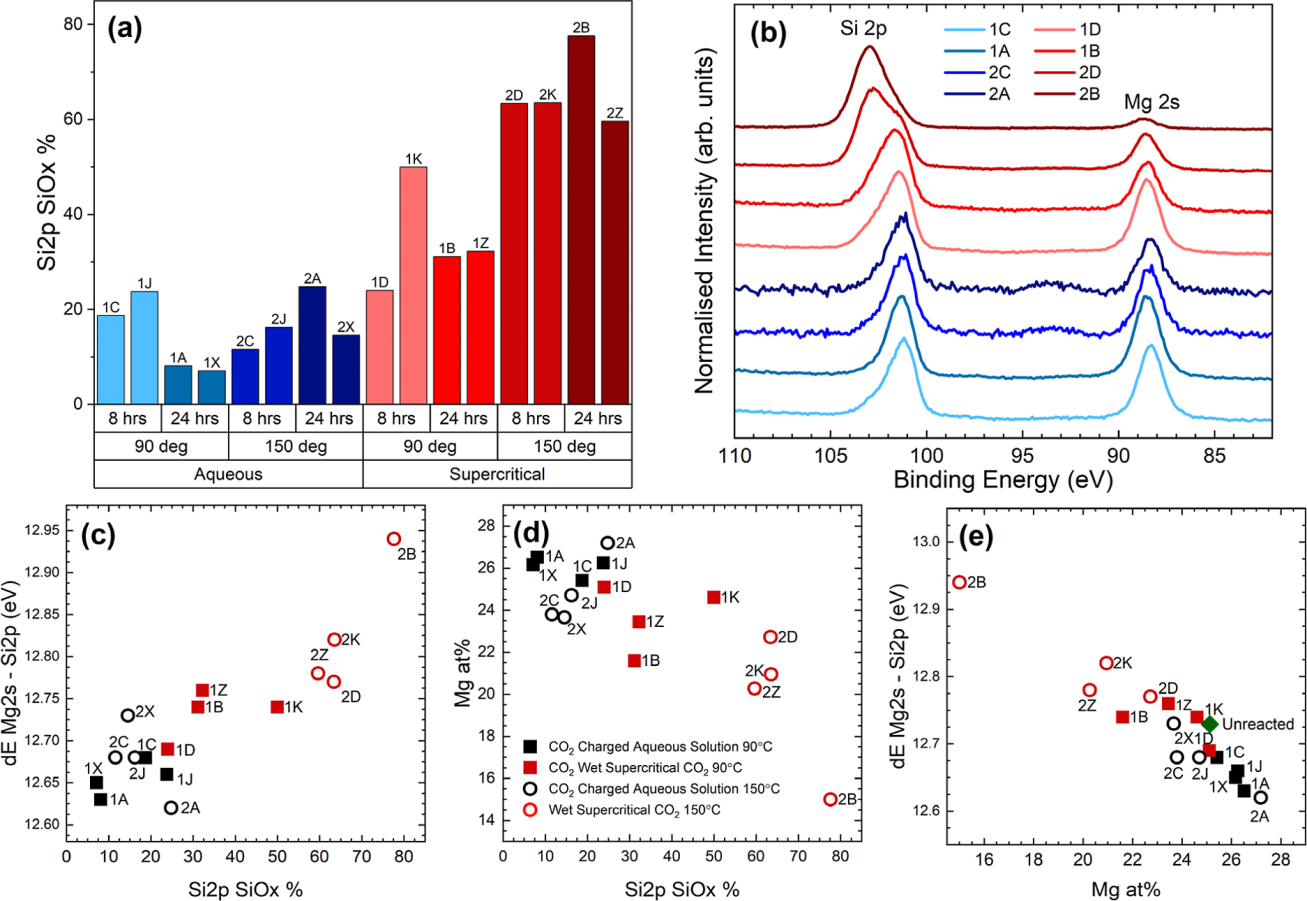
Plots of XPS quantities obtained for each of the 16 dissolution experiments showing (a) level of SiO_*x*_ formation under different combinations of reaction conditions. No SiO_*x*_ was detected in unreacted reference samples; therefore, there is no data point to add. (b) Normalized XPS data showing the intensity of the Mg 2s in comparison to both Si 2p components across all reaction conditions [the same color code is maintained as in part (a)], (c) binding energy separation between Si 2p and Mg 2s transition peaks (in olivine) vs SiO_*x*_ concentration, (d) Mg atomic percentage (in olivine) vs SiO_*x*_ concentration, (e) binding energy separation between Si 2p and Mg 2s vs Mg atomic percentage (in olivine) compared against a reference unreacted sample data point (average of 14 separate unreacted olivine sections). All binding energy separations and area ratios only consider the core level components attributed to remaining olivine.

Examination of the Si 2p core levels reveals a significantly greater intensity of SiO_*x*_ in scCO_2_-reacted surfaces than that in aqueous-reacted surfaces (Figure 6 comparing panels a–d for aqueous experiment data, to panels e–h showing scCO_2_ experiment data). It is noted that this increased intensity could indicate either a greater thickness of the SiO_*x*_ layer or a greater coverage. SiO_*x*_ quantities extracted from these core level fitting data are presented as percentages of the Si 2p peak area (Table 4). Due to the inherent surface sensitivity of XPS, and the dependence of this surface sensitivity on the binding energy of the core level in question, it is often not practical to perform quantitative analysis of composition using this technique. However, due to the similar binding energy of the Si 2p (∼100 eV) and Mg 2s (∼89 eV), the surface sensitivity of the measurement to the two elements is similar for these specific peaks. Given this context, further quantitative conclusions can be drawn from the XPS data. Quantities of SiO_*x*_ detected in areas sampled from the reacted olivine surfaces are presented in Table 4 as a percentage of the total Si signal in the near-surface region. Mg/Si data obtained from core levels corresponding to olivine are also included, showing decreased levels of Mg in highly reacted surfaces. Key trends are listed in Figure 7.

**Table 4:** Intensity Ratios and Binding Energy Separations Derived from XPS Core Level Fitting Data^a^

sample code	CO_2_ condition	reaction time (h)	SiO_*x*_ content (% area of Si 2p peak)	Mg/Si ratio (Si in silicate)	Mg 2s–Si 2p binding energy separation (eV)	Si 2p (silica–silicate) binding energy separation (eV)
Unreacted						
				1.41:1	12.73	
Subsurface Reaction Conditions (90 °C)						
**1C**	aqueous sol	8	23.8	1.57:1	12.66	1.48
**1J**	aqueous sol	8	18.7	1.44:1	12.68	1.42
**1A**	aqueous sol	24	8.1	1.61:1	12.63	1.52
**1X**	aqueous sol	24	7.1	1.34:1	12.65	1.57
**1D**	wet scCO_2_	8	50.0	1.34:1	12.74	1.29
**1K**	wet scCO_2_	8	24.0	1.40:1	12.69	1.39
**1B**	wet scCO_2_	24	29.7	1.01:1	12.74	1.17
**1Z**	wet scCO_2_	24	32.2	1.20:1	12.76	1.30
Enhanced Reaction Conditions (150 °C)						
**2C**	aqueous sol	8	11.6	1.23:1	12.68	1.54
**2J**	aqueous sol	8	16.2	1.35:1	12.68	1.44
**2A**	aqueous sol	24	24.8	1.83:1	12.6	1.27
**2X**	aqueous sol	24	14.5	1.26:1	12.73	1.55
**2D**	wet scCO_2_	8	63.4	1.12:1	12.77	1.38
**2K**	wet scCO_2_	8	63.6	0.95:1	12.82	1.22
**2B**	wet scCO_2_	24	77.6	0.54:1	12.94	1.26
**2Z**	wet scCO_2_	24	59.6	0.89:1	12.78	1.33

a Note: unreacted data are obtained by averaging data from 14 separate specimens.

Enhanced silica signals are apparent in all scCO_2_-reacted samples including the samples which have not exhibited any observable silica precipitation, e.g., those reacted in 90 °C, 9 MPa conditions for 8 h (Table 4). The four highest SiO_*x*_ concentrations occur exclusively in samples reacted in wet scCO_2_ at 150 °C. Only one other sample exhibits SiO_*x*_ formation over 40%, which is also reacted in scCO_2_. To exemplify this trend, SiO_*x*_ percentages measured from all reacted samples were grouped according to the environment, temperature, and duration (Figure 7a). By contrast, aqueous-reacted samples show very low levels of silica formation, and no trend is apparent. In fact, the highest silica levels among aqueous-reacted samples are measured under the 8 h, 90 °C conditions (i.e. the shorter duration and lower temperature experiments). The low SiO_*x*_ levels observed in these samples relative to scCO_2_-reacted olivine crystals, coupled with the presence of surface salt deposits, introduce a higher degree of uncertainty associated with the aqueous-reacted sample measurements, potentially accounting for this counterintuitive result. Alternatively, Si release into the bulk solution via silica redissolution is a possibility, especially under aqueous conditions.

The effect of temperature (and reaction time) on surface chemistry is evident in wet scCO_2_ reaction systems (Figure 7). Increasing the system temperature from 90 to 150 °C greatly promoted SiO_*x*_ formation in the CO_2_-rich system relative to the water-rich system. A corresponding decrease in magnesium peak intensity is observed with the transition from water-rich to CO_2_-rich environments and with increasing system temperature (Figure 7b,d). It is also noted that increased SiO_*x*_ levels correspond with an increase in the energy separation between the Si 2p and Mg 2s peaks (Table 4) (Figure 7c), attributed to Mg depletion in the underlying olivine layer (Figure 7e).

The XPS data presented here show greater reactivity of forsteritic olivine in water-saturated supercritical CO_2_ as compared with CO_2_-saturated water, for the same pressure, temperature, and reaction time. In the following, we also provide added support for this from reaction textures imaged on the surface of the minerals.

### 3.3 Reaction Textures and Product Layers

**Figure 8:**
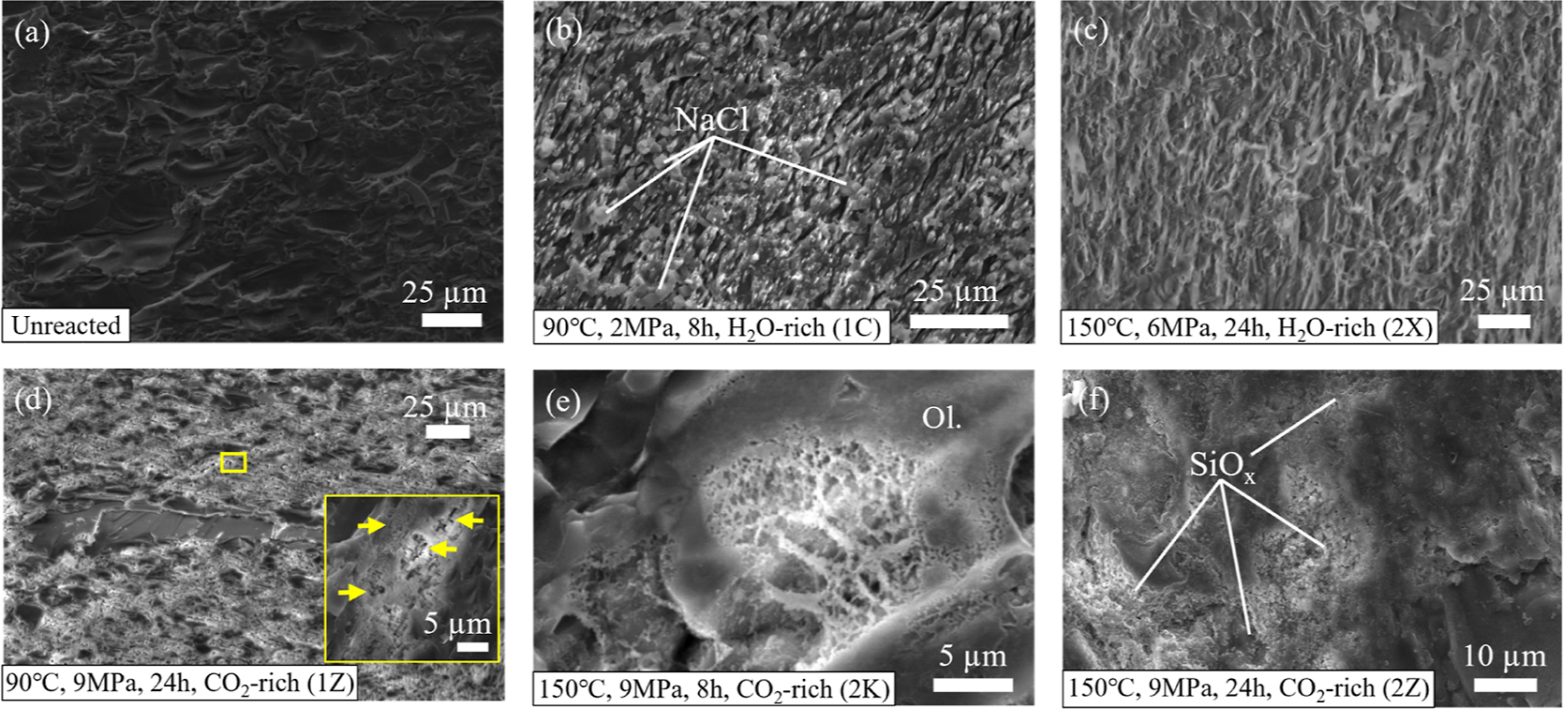
Olivine surface textures observed under SEM of (a) reference unreacted sample displaying only the elongated grooves originating from sample preprocessing, (b,c) samples reacted in water-rich environments under (b) reservoir and (c) enhanced conditions showing no discernible difference in surface texture compared to unreacted samples apart from the presence of salt deposits, and (d–f) samples reacted in CO_2_-rich environments showing different dissolution reaction etches (etch-pits): (d) on surfaces absent of precipitates and (e) on surfaces which produced minor fragmented precipitates (see Figure 9a,b). (f) Pervasive Si-rich dissolution byproduct formations which dominated highly reacted regions of the olivine samples reacted in 150 °C, scCO_2_ conditions for 24 h.

In accord with PHREEQC models (Figure 5b,c), SEM analysis revealed no Si-rich precipitates formed on mineral surfaces reacted in water-rich experiments under 90 °C (2 MPa) and 150 °C (6 MPa) conditions. Additionally, negligible differences in surface texture were observed between the unreacted samples (Figure 8a) and those that reacted with an aqueous solution (Figure 8b–c).

The only secondary minerals identified by SEM-EDXS in this study are silica precipitates formed under the most aggressive reaction conditions, at 150 °C in scCO_2_ lasting 8- and 24 h (Experiments 2D, 2K, 2B, and 2Z). SEM analysis of sample surfaces exposed to wet scCO_2_ at 90 °C also showed no silica precipitates, despite the fact that XPS data indicated a stronger SiO_*x*_ signal (Figure 6e,f). However, extensive dissolution was evident from the reaction textures (dissolution etch-pits) observed across the olivine surface (Figure 8d), akin to those imaged in 150 °C CO_2_-rich experiment samples (Figure 8e). Conversely, reacted regions of surfaces in 24 h 150 °C CO_2_-rich experiments exhibited extensive surface product coverage (Figure 8f).

These observations show that the trends in the extent of magnesium-depletion at the mineral surfaces observed in XPS occurs irrespective of the existence of a reprecipitated silica layer. For the silica products that occurred in experiments 2D, 2J, 2B, and 2Z, it is not possible to identify whether they formed during the controlled reaction period, or whether they formed as the sample was depressurized, cooled, and dried. Thus, they could be indicative of the saturation state being achieved in a thin film of water on the surface of the minerals or limitations to the transport of dissolution products away from the reaction site resulting in precipitation as the sample was retrieved. However, in the circumstance that layers formed during the controlled reaction conditions themselves, they are of considerable interest, and we report further analyses of these layers in the following paragraphs.

**Figure 9:**
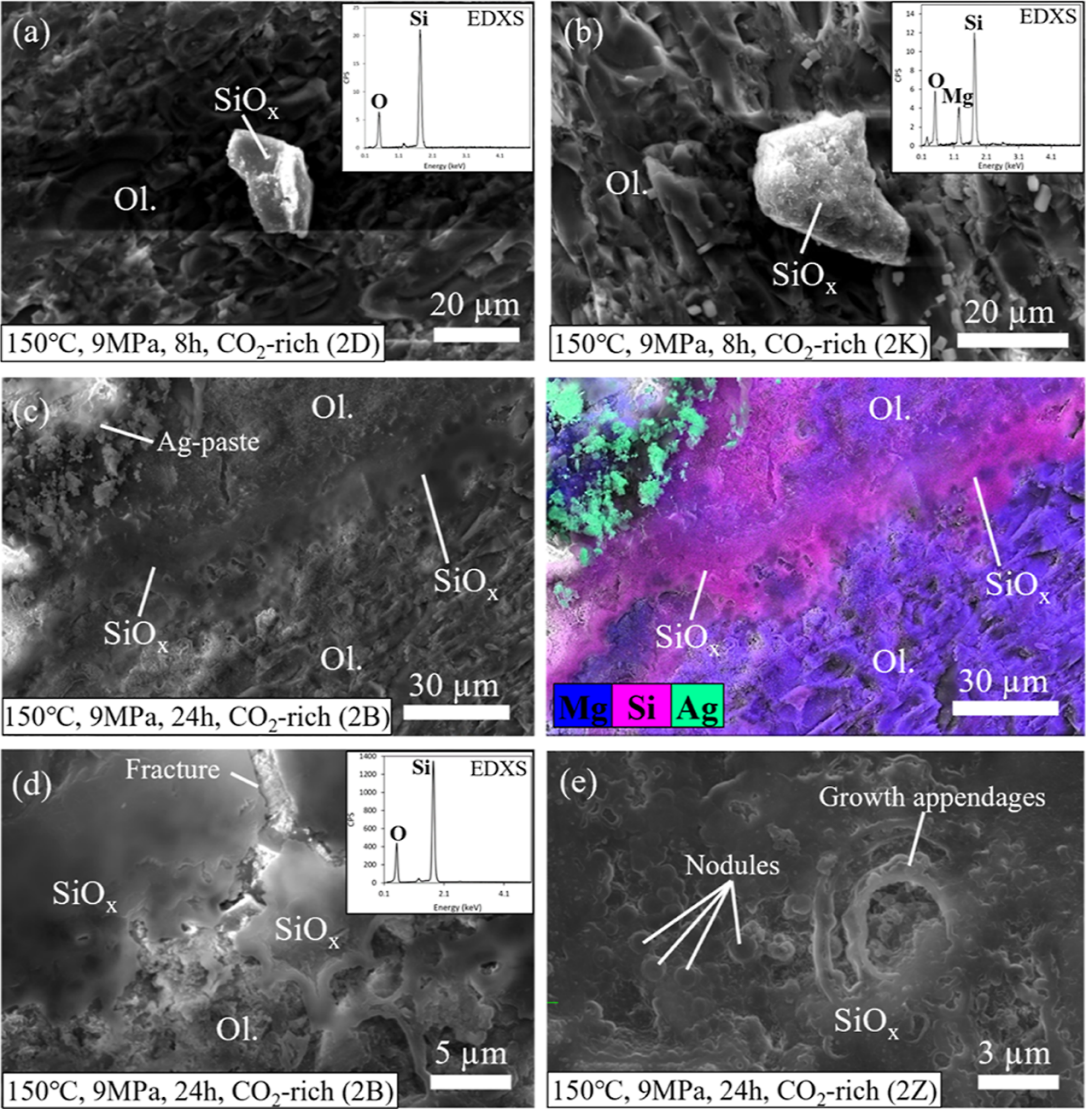
SEM-EDXS analysis highlighting the contrasting style of silica product formation on samples reacted in 150 °C wet scCO_2_ systems for 8 and 24 h. Note: EDX spectra shown in (a,d) are obtained from sections labeled SiO_*x*_. (a,b) Isolated Si-rich products formed on samples reacted for 8 h (2D and 2K, respectively), (c) Si-rich coating and complementary EDX elemental map of the equivalent area in sample 2B reacted for 24 h (the presence of silver is due to the addition of silver paste during SEM preparation), (d) magnified image showing smooth topography of Si-rich coatings relative to the rough olivine surface and cracks across the silica coatings, (e) concentric growth features embedded within Si-rich layers formed in sample 2Z reacted for 24 h.

Increasing temperature to 150 °C resulted in the formation of sporadically distributed micrometer-scale (20–40 μm) Si-rich fragments (Figure 9a,b). The bulk composition EDXS analysis of these phases indicates minor traces of magnesium. Prolonging reaction time to 24 h while maintaining 150 °C reaction conditions in scCO_2_ experiments resulted in the formation of silica layers spanning tens of micrometers in length (Figure 9c–e). Formation of these coatings occurred preferentially near the sharp edges of the samples. The presence of an EDXS signal consistent with a SiO_*x*_ product confirmed that these silica precipitates were at least 1 μm thick (Figure 9c,d). Pervasive precipitates were observed solely under these reaction conditions and represent an advanced stage in the growth of Si-rich precipitates across the mineral surface.

**Figure 10:**
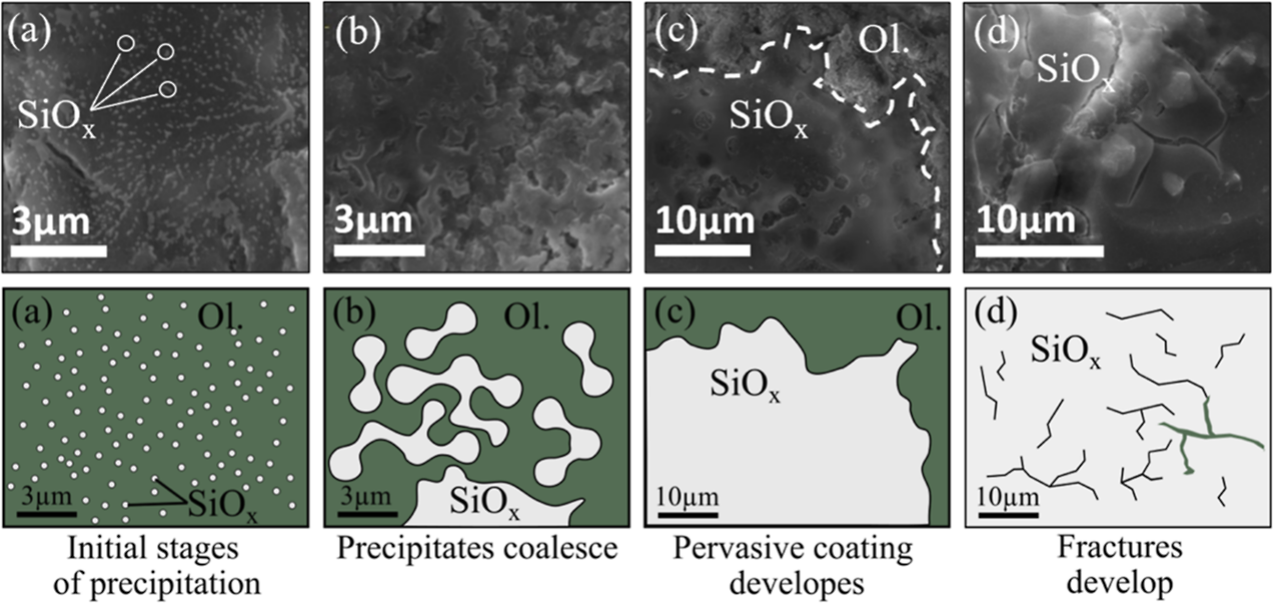
SEM–EDX images exhibiting four distinct stages in silica passivating layer growth (a–d) taken from different areas across the planar surfaces of samples 2B and 2Z (reacted in 150 °C wet scCO_2_ systems for 24 h). Complementary schematics are provided adjacent to gray scale SEM images to accentuate the key features of each silica growth stage observed on olivine surfaces.

The array of SiO_*x*_ microstructures reveals distinct stages in the development of silica layers across the olivine surface (Figure 10). Initially, nucleation sites emerge as approximately 200 nm growth centers scattered across the surface (Figure 10a). These nucleation zones (nodules) coalesce, resulting in a thickened mass at the reactive surface (Figure 10b,c). A common late-stage characteristic of the coatings is the propagation of brittle fractures (Figure 10d). Fractures extend through the entire thickness of the layers, exposing the underlying olivine surfaces, and are seen to lengthen with time postreaction. These outcomes highlight a spatiotemporal evolution of mechanical passivation across the planar reactive mineral surface in the wet scCO_2_ reaction domain.

### 3.4 Cross-Sectional Analysis of the Precipitate Layer

**Figure 11:**
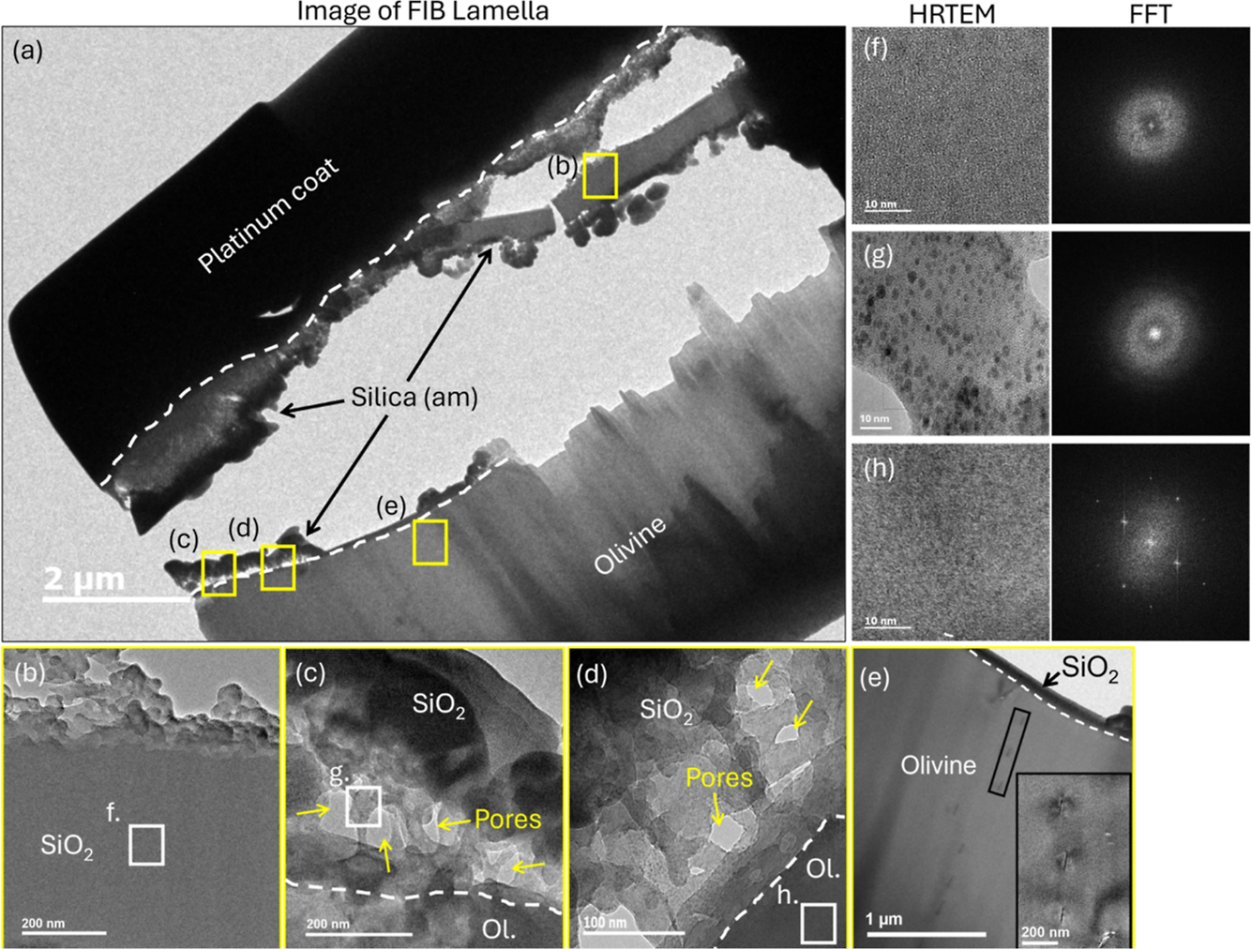
(a) TEM cross-section image of FIB lamella obtained from the pervasive silica reprecipitate layer observed in Figure 9d formed in experiment 2B (150 °C, 9 MPa, wet scCO_2_). Sections of the silica layer (arrowed) partitioned from the underlying olivine during thinning down of the FIB lamella. The original upper and lower bounds of the reaction layer are traced with a dashed white line. (b–d) Close-up images showing structural features and porosity (arrowed) within the passivating silica layer. (e) Image of lattice strain (black outline) indicated by the defects in olivine crystal structure which do not correspond to any variation in chemical composition and extend away from the olivine–silica interface (dashed line). (f–h) HRTEM of (f) bulk silica phase, (g) pore throats, and (h) crystalline olivine substrate (intraband distance of 0.3658 nm). Adjacent to these HRTEM are complementary FFT analysis of the three points across the interfacial region highlighting the shift from (f,g) amorphous silica to (h) crystalline olivine material. Note: dark spots visible in HRTEM images correspond to gallium nanocrystals released during milling of the lamella.

**Figure 12:**
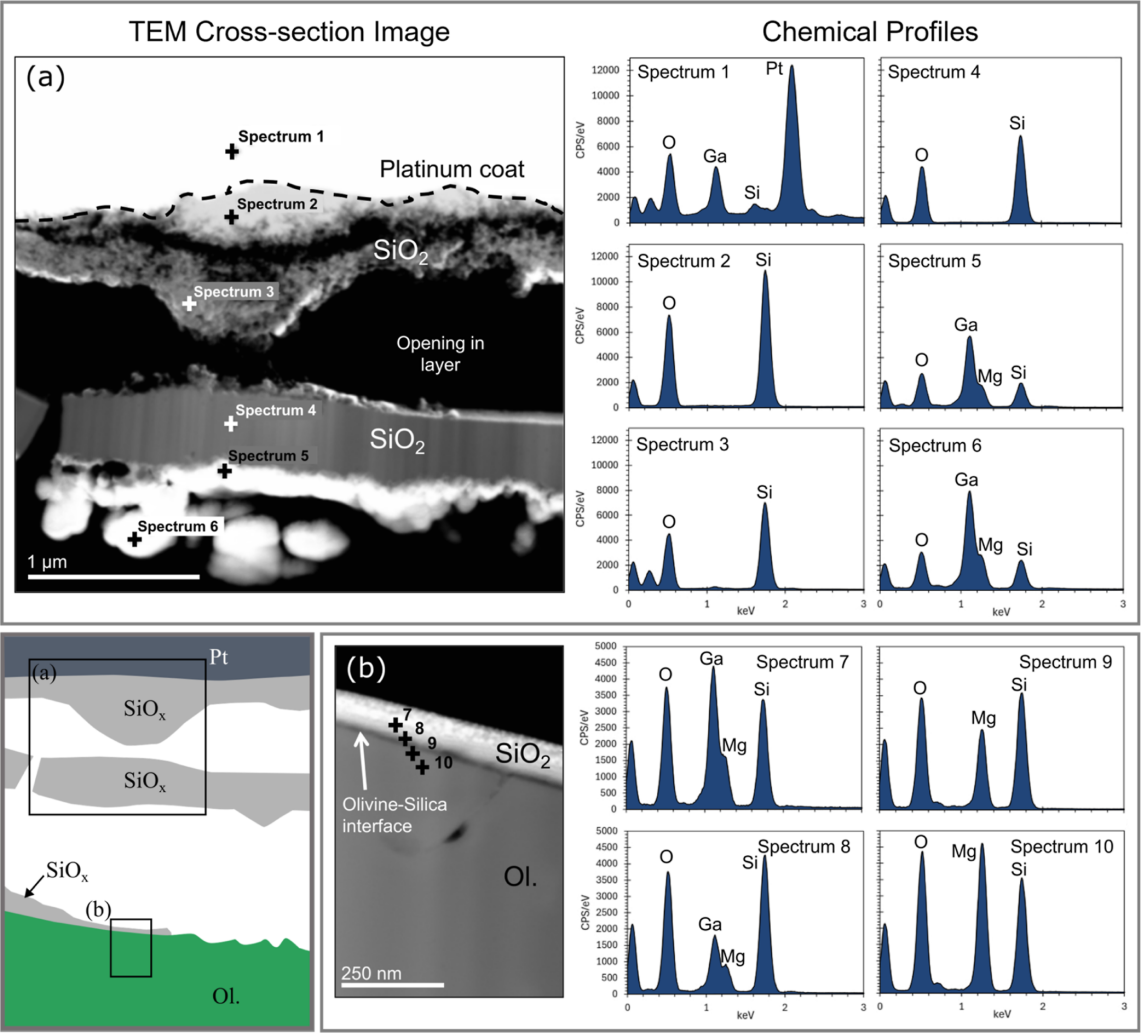
TEM–EDX chemical profile analysis across the silica layer formed from experiment 2B. A schematic diagram illustrating the respective positions of each TEM cross-section image taken from the FIB section is shown. (a) Upper section of the amorphous silica (including the platinum coating used for FIB milling) also shown in Figure 11b. (b) Lower section of the silica layer (same location as shown in Figure 11e). The reaction surface is represented by the dashed line. EDX spectra data were collected at intervals across the platinum coat (spectrum 1), through the silica layer (spectra 2–8), and in the underlying olivine substrate (spectra 9 and 10) and show the gradual emergence of the magnesium peak with depth across the amorphous silica.

TEM, STEM, and EDX analyses were conducted on samples from experiments 2B (150 °C, 9 MPa, 24 h, scCO_2_) (Figures 11 and 12) to investigate the structural and chemical properties of the reaction layer formed at the mineral–fluid interface during dissolution. High-resolution TEM (HRTEM) images were further analyzed using fast Fourier transformation (FFT) to show lattice arrangements in the interfacial regions. Results outlined in this section provide insights into passivation effectiveness and the physical stability of silica layers formed in wet–supercritical reaction environments.

The FIB lamella produced from sample 2B shows three distinct phases (Figure 11a). A platinum protective coat applied prior to FIB milling overlies the silica layer (outlined), which was partially separated during sample thinning stages (Figure 11b). A well-defined contact surface separates the silica from the underlying olivine phase (Figure 11c–e). FFT analysis of the interfacial structures shows a clear deviation from the silica layer, which displays a characteristic disordered (amorphous) texture (Figure 11f–g) to the periodic lattice arrangement of olivine (Figure 11h). Regions of the silica layer closest to the olivine substrate display an extensive porosity (Figure 11c,d). Pore diameters (parallel to beam) range in size from 50 to 100 μm with narrower vertical pores also present. Analysis of pore throats confirms an amorphous silica phase (Figure 11g) that incorporates traces of magnesium (see Figure 12). Conversely, the upper regions of the silica layer show a lack of extensive porosity (Figure 11b). The olivine appears structurally compromised beneath the interface with the silica. Nanometer-scale defects (lattice strains) were imaged penetrating up to 2 μm into the olivine crystal (Figure 11e) and showed no changes in chemical composition.

Closer inspection of the reaction layer chemistry via TEM-EDXS draws attention to a clear trend in the magnesium content with depth across the amorphous silica layer. The upper portions of the layer exhibit little to no magnesium signal (Figure 12a). However, EDX spectra taken at intervals farther away from the original silica–fluid interface show a gradual appearance and development of the magnesium peak down to the original olivine substrate (Figure 12b). The regions where magnesium begins to appear do not correspond to crystallinity; the amorphous structure persists throughout the silica layer until the bulk olivine phase is reached.

## 4 Discussion

### 4.1 Reaction Progress across Reaction Systems

The formation of precipitates alone cannot reliably indicate differences in reactivity between water-rich and CO_2_-rich environments. This is because reactions in these environments involve significantly different volumes of water. Therefore, complementary nanometer-scale analyses of the interfacial region using XPS are necessary to discern differences in reactivity more accurately.

Our results demonstrate that silicon enrichment alongside magnesium depletion trends in XPS data depend primarily on the fluid phase and second upon the temperature at which reactions were conducted (Table 4). Improved reactivity is evident in the CO_2_-rich fluid relative to the water-rich mixtures based upon the increased silica concentrations derived from XPS analysis. The increased SiO_*x*_ signal is evident in scCO_2_-reacted samples relative to aqueous counterparts even in the absence of precipitates (Figure 6a–f) (Table 4) and is attributed to the degradation of the silicate structure as magnesium is extracted, leaving a silica-rich leached layer at the surface, which subsequently breaks down into silica monomers. Detection of the SiO_*x*_ signal in samples which have not formed densified precipitates can therefore imply that silica monomers have not repolymerized under conditions specific to those experiments.^28^ The presence of precipitates in 150 °C wet scCO_2_ experiments greatly contributes to the overall SiO_*x*_ concentration (Figures 6g,h and 7a). Consistent with this trend, XPS data indicate that the highest rate of SiO_*x*_ formation occurred in experiment 2B (24 h, 150 °C, wet scCO_2_), with 77.6% of the detected Si attributed to SiO_*x*_.

The dependence of the SiO_*x*_ concentration (and thereby reactivity) on the temperature conditions of the experiment is clearly exhibited in our data (Figure 7a). This relationship is particularly evident in wet scCO_2_-reacted samples. The effect of temperature on the kinetics of the dissolution reaction is exemplified in our modeling of the reaction taking place in a water-rich environment and will promote the extent of reaction as well in wet scCO_2_. However, the solubility of water (mole fraction of H_2_O) in the CO_2_-rich phase also increases exponentially with temperature, nearly doubling from 90 to 150 °C at a constant pressure of 10 MPa,^24^ and this may also be a driver of increased reactivity. Given the critical role of water in facilitating the dissolution reaction of olivine in CO_2_-rich fluids, increasing the system temperature leads to pronounced changes in reactivity in the supercritical domain as is exhibited in all data sets presented in this work.^25^

The pattern of silica enrichment in wet scCO_2_-reacted olivine is complemented by reductions in near-surface Mg signatures compared with aqueous-reacted samples and the unreacted reference data point (Figures 7b,d–e, S4) (Table 4). Depletion of Mg from the olivine surface during dissolution is also accompanied by a shift in the core level binding energy (Figure 7e) and a greater binding energy separation between the Si 2p (olivine) and Mg 2s peaks. This is consistent with a reduced electron density around the Si nuclei and further supports the presence of a layer depleted in magnesium (see Table S1 for full list of binding energies).^29^ Data from wet scCO_2_ runs show that near-surface olivine becomes increasingly Mg-poor particularly when reacted under enhanced temperature conditions (Figure 7b). These trends are particularly pronounced in samples where pervasive amorphous silica coatings formed, reaching a minimum of 15 at. % Mg (Figure 7d). Conversely, samples reacted with a water-rich fluid exhibit surface chemical signatures comparable to the average of unreacted reference samples (a less pronounced SiO_*x*_ signal and high Mg intensity), indicating reduced reactivity, shallower reaction penetration, and less effective metal leaching from the olivine substrate. Given the expectation of limited silica precipitation in water-rich experiments, the depletion of Mg serves as an additional metric for comparing dissolution progress across various fluid environments.

### 4.2 Reactant Transport and Passivation Effectiveness across Reaction Systems

Surface passivating silica was only produced on olivine reacted in CO_2_-rich systems under enhanced temperature and pressure conditions (150 °C, 9 MPa) and indicate limited reactant transport from reaction sites. Supersaturation with respect to silica was not predicted from PhreeQC simulations and was not observed during reaction time under 90 °C experiments in water-rich reaction environments (Figure 5b,c). Counterpart experiments conducted at 90 °C in CO_2_-rich systems also revealed no evidence of silica precipitation detectable by SEM. These results imply negligible mechanical passivation of reactive surfaces at reservoir conditions during our reaction timeframes. Conversely, 150 °C scCO_2_ experiments lasting 8 h resulted in silica precipitation (Figure 9a,b). The scattered precipitates formed under these CO_2_-rich conditions suggest local states of supersaturation were achieved within a water medium accumulated at the mineral surface, but it is not possible to definitively state whether this occurred during the experimental time frame or while the samples were brought back to atmospheric conditions. Evaporation of the adsorbed water film may have occurred during system depressurization and subsequently facilitated supersaturation with respect to silica. The concept of a shrinking water film is supported by the formation of NaCl ring precipitates exclusively across wet scCO_2_-reacted surfaces (see Figure S3). In either case, water films on the hydrophilic olivine surface are the location where fluid-mineral reactions occur.^18,30^ The localized precipitates are indicative of limits to the transport of reactants, e.g., dissolved silica, away from reaction sites. Flow of the water film across the reactive interface, as would be the case in reactive porous media, is therefore important in facilitating the continued dissolution of the mineral phase.

The effect of reaction time on the surface chemistry of reacted olivine is not discernible in the XPS data. This is likely because the reaction does not alter bulk chemical expression of the interface to within 10 nm depth, (across a sample area of 0.13 mm^2^) enough from 8 to 24 h reaction periods, particularly in samples reacted in water-rich mixtures. However, mapping the development of silica layers with varying reaction time scales using SEM–EDX reveals a shift in passivation effectiveness with time in addition to temperature in the supercritical CO_2_ domain. Prolonging reaction time to 24 h in 150 °C and 9 MPa in wet scCO_2_ environments resulted in a substantial contrast in style of product formation. Figure 9 exemplifies this shift toward pervasive Si-rich coatings. TEM analysis of the silica–olivine interface (Figure 11c–e) reveals direct coverage of the reactive surface across these regions. This implies a marked shift in surface coverage and passivation effectiveness of the underlying substrate compared with 8 h-reacted counterparts. Second, it signifies that the threshold for supersaturation with respect to silica was met during reaction time within the confined volume of water formed at the surface under 150 °C, contrary to water-rich or 90 °C wet scCO_2_ experiments. The rapid expansion of reaction products with time in wet scCO_2_ can be explained by the absence of bulk water properties mitigating redissolution of these phases, in turn enabling the growth of silica products with time.

Cross-sectional analysis of amorphous silica structures indicates extensive reprecipitation of passivating SiO_*x*_ groups during dissolution and shows leached metal ions are incorporated into the reprecipitate. A clear magnesium concentration gradient is present through the reaction layer, increasing with the proximity to the olivine substrate (Figure 12). This corresponds with an observed increase in layer porosity (Figure 11) and may suggest that leached magnesium disrupts the polymerization of silica during reprecipitation and reduces its passivation effectiveness by compromising the sealing efficiency of the layer. Metal ion incorporation into the silica layer has also been identified as a potential cause of silica structure distortion by Daval et al. (2009).^31^ However, the water content of the system also influences the microstructure of the product layer and subsequently the reaction extent.^32^ Consequently, the methodology employed by Daval et al. (2009) does not strictly regulate the quantity of water within the system, as reactions in both water-rich and water-bearing supercritical CO_2_ environments were conducted simultaneously in a single vessel.^31^ In contrast, the experimental setup used in our study guarantees precise control over the water content in both solvent systems, as the experiments are conducted independently. This enables our results to provide more representative conclusions regarding passivation in wet scCO_2_ systems. The presence of pore space within passivating layers is essential for allowing the initially nonstoichiometric dissolution of olivine and sustaining the release of metal cations needed for carbonation.^21^ However, efficient silica polymerization and reprecipitation across the surface in scCO_2_ reaction environments presents a clear resistance to mass transfer of reactants required for carbonate formation further along the mineralization pathway.

Brittle fracturing of the product layer and the straining of the underlying olivine substrate are secondary effects of the dissolution reaction. The susceptibility of the passivating layer to brittle fracture is evident in scCO_2_-reacted samples (Figures 9d and 10d). This effect stems from the high tensile stress caused by the discrepancy in molar volume as silica forms from the olivine phase and may reduce the mass transfer resistance posed by the silica.^8,21^ Furthermore, the formation of a silica phase in the early stages of CO_2_-induced olivine dissolution invokes strain on the underlying olivine, resulting in the propagation of fractures from the olivine–silica interface (Figure 11e). Evidence of lattice strain were observed to micrometer-scale depths and could represent the nucleation of deep-set fracture networks within the reactive mineral phase. This observation underscores the importance of the dissolution reaction in potentially promoting fracture-driven in situ mineralization within the pore spaces.^33^ The enhanced silica production observed in wet scCO_2_ systems and the resulting volume contraction across the reactive interface may expedite fracture formation in the parent mineral and potentially positively impact the overall extent of mineralization.

## 5 Conclusions and Implications

This study examined the effects of CO_2_-induced forsteritic olivine dissolution at the mineral–fluid interface in both water-rich and CO_2_-rich reaction systems under equivalent experimental conditions. The selection of olivine provides a simplified model of the overall reactivity in basaltic rock and serves as an analogue for reactive mineral interfaces in the presence of CO_2_-rich fluids. The planar surfaces of olivine crystals provide a valuable intermediate between powder-based experiments and the reactive surfaces found in the millimeter-scale vesicles and planar fractures of olivine-rich basaltic flow-top reservoirs in CarbFix and Wallula. Additionally, the single-crystal setup prevents mechanical disturbances and allows for spatial resolution of the product nucleation across the reactive surface. Hence, the sample geometry presented here provides a more accurate model of surface mechanical passivation on the surfaces of pores or fractures in mafic rock formations compared to powder-based studies.

We show that silica layers formed during olivine dissolution in CO_2_-dominated fluids exhibit clear cross-sectional chemical variations, with leached metal cations, particularly magnesium, becoming incorporated into the passivating silica phase. This early trapping of magnesium alters the porosity and impacts the effectiveness of the passivation layer, potentially hindering its availability for later carbonation reactions. The silica layers, which fractured and exfoliated without surface agitation, are potentially associated with mechanical degradation of the underlying olivine substrate. These findings are crucial for understanding how reactants required in facilitating CO_2_ mineralization are transported across the mineral-fluid interface within subsurface reservoirs where CO_2_-rich and water-rich reaction systems coexist.

While previous studies have examined reactivity in wet supercritical CO_2_, our research is the first to rigorously compare reaction extents, showing that olivine dissolves more readily in water saturated CO_2_-rich fluid than that in CO_2_-saturated water-rich fluid systems at the same temperature and pressure. Thus, we propose a higher dissolution rate in water-bearing supercritical CO_2_ environments. This is evidenced by SEM–EDX and XPS data, which reveal a greater quantity of surface silica, less Mg, and greater dissolution front penetration into the olivine substrate in wet scCO_2_-reacted samples compared to aqueous counterparts at equivalent conditions. These alterations become pronounced under elevated temperature and pressure, with implications for optimizing surface-engineered mineralization operations. The potential use of alternative CO_2_-rich fluid chemistries could lead to more cost-effective mineral trapping.

Our results imply that the overall rate of CO_2_ mineralization in geologic reservoir formations comprises two distinct reaction regimes stemming from the fluid environments that evolve within the pore space upon CO_2_ injection. The total reaction rate in a subsurface system where both fluids exist may be dominated by the rate in the supercritical CO_2_. This would in turn suggest that there may be some advantage to subsurface injection schemes where free phase CO_2_ is injected (as at Wallula) relative to schemes where water saturated in CO_2_ is the injection fluid. However, this benefit would have to be balanced against any increased risk to CO_2_ leakage to the surface from the buoyant nature of the free phase CO_2_. These findings highlight the potential for optimizing CO_2_ injection strategies to enhance long-term storage stability.

Future work should focus on quantifying the rate at which reactions take place in CO_2_-rich fluid phases, incorporating more complex sample geometries and mineralogy, and integrating batch reactor studies with core-flooding experiments. These efforts will offer deeper insights into fluid-mineral interactions under dynamic conditions and help optimize the balance between dissolution rates and long-term stability of the reactive interface in natural geologic systems.

## Supporting Information Available

Additional experimental details: sample image, EDX spectra, XPS, and SEM imagery (PDF)
