# Supplementary material for: Enhanced Olivine Reactivity in Wet Supercritical CO2 for Engineered Mineral Carbon Sequestration
Source: Energy Fuels. 2024 Oct 28;38(21):21028–41. doi: 10.1021/acs.energyfuels.4c04120 (PMC11551954; doi:10.1021/acs.energyfuels.4c04120)
Supplement: Supplementary file 1 — ef4c04120_si_001.pdf [file ef4c04120_si_001.pdf]

Enhanced Olivine Reactivity in Wet Supercritical CO_2 for Engineered Mineral Carbon Sequestration

Mohamed A. Saleh [a*], Huw Shiel [c], Mary P. Ryan [c], J.P. Martin Trusler [b] and Samuel Krevor [a]

[a] *Department of Earth Science and Engineering, Imperial College London, Exhibition Road, London SW7 2AZ, United Kingdom.*

[b] *Department of Chemical Engineering, Imperial College London, Exhibition Road, London SW7 2AZ, United Kingdom.*

[c] *Department of Materials Science, Imperial College London, Exhibition Road, London SW7 2AZ, United Kingdom.*

mohamed.saleh18@imperial.ac.uk,h.shiel@imperial.ac.uk,m.p.ryan@imperial.ac.uk,m.trusler@imperial.ac.uk , s.krevor@imperial.ac.uk

Supplementary information sheet

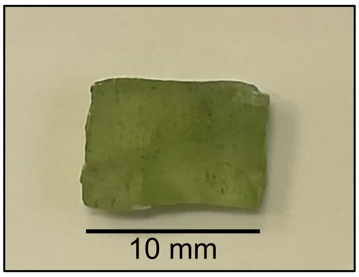

Figure S1. Example forsteritic olivine crystal section cut. Note: Dark impurities are characterised as diopside (see fig. S2).

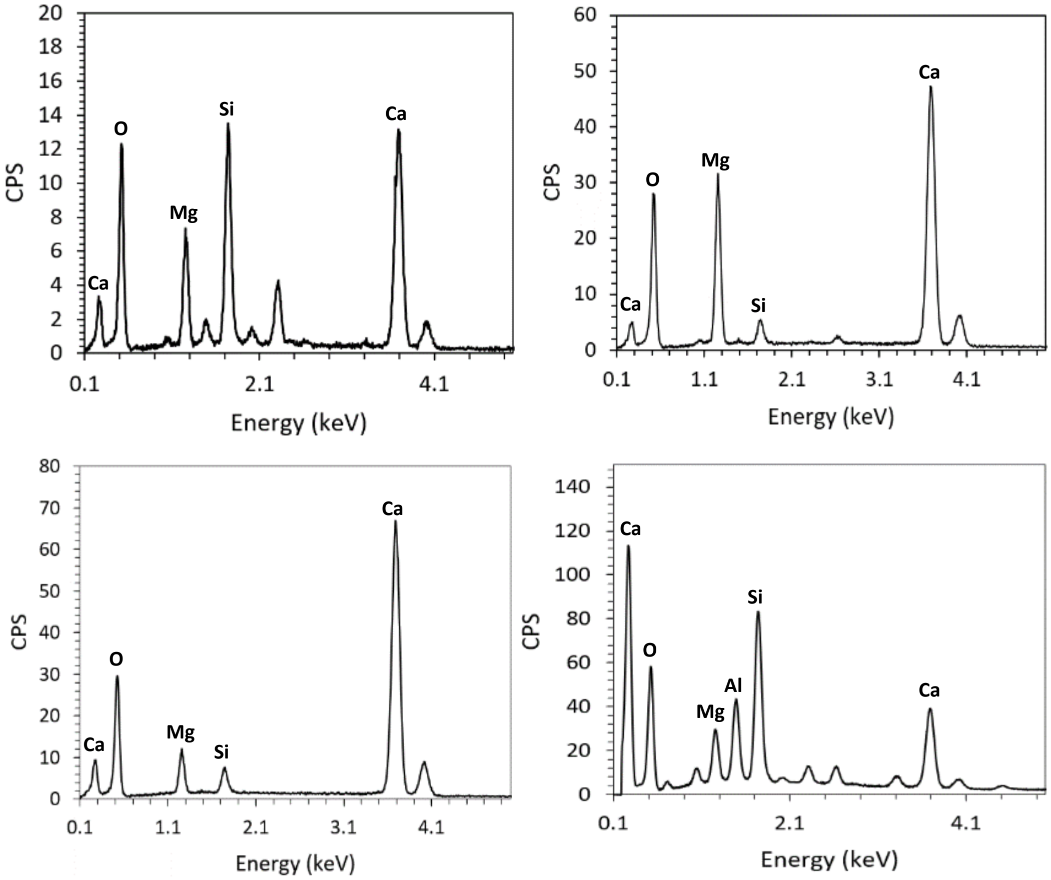

Figure S2. Example EDX spectra of Al-Diopside impurities characterised by Ca content and found embedded on the olivine surfaces.

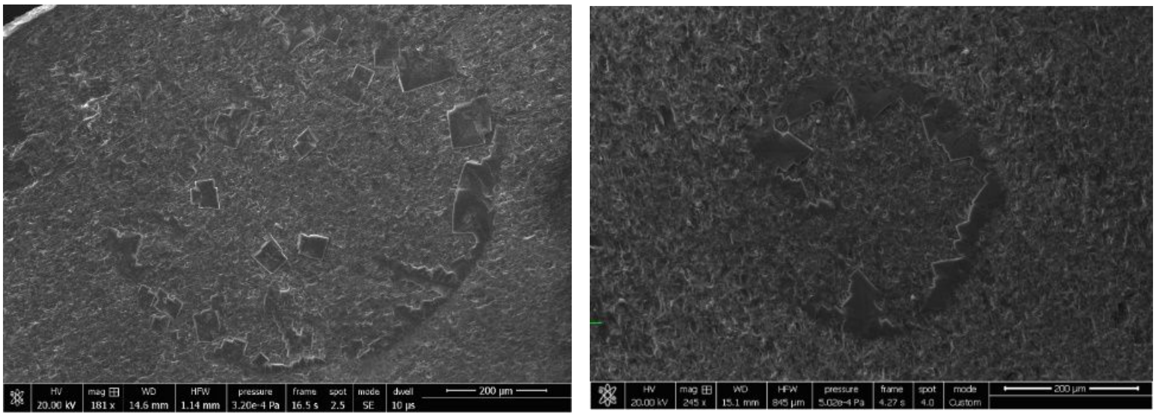

Figure S3. Salt precipitate rings formed on separate olivine samples reacted in wet supercritical CO_2.

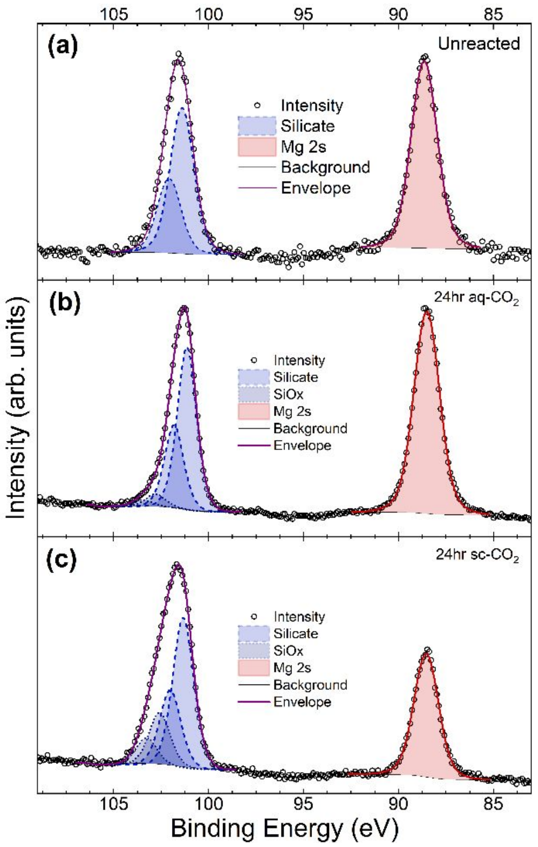

Figure S4. XPS data from samples (a) Unreacted, (b) SB-1A and (c) SB-1B, showing the proximity of Mg 2s peak to Si 2p peak. It is evident from inspection of the data that the intensity of the Mg 2s is lower in comparison to both Si 2p components in SB-1B. The full set of intensity ratios extracted from all samples is included in Table 4 in the main manuscript.

Table S1. Supplementary XPS data

Sample code	Si 2p 3/2 Binding Energy (eV)	Si 2p 3/2 Area (arb. units)	Si 2p 3/2 Binding Energy (eV)	Si 2p 3/2 Area (arb. units)	Mg 2s Binding Energy (eV)	Mg 2s Area (arb. units)
	Silicate Contribution		SiO_x Contribution		Silicate Contribution	
Unreacted (average)	101.35	7777.84	N/A	N/A	88.62	7605.44
SB-1A	101.14	10295.65	102.66	907.473	88.51	11588.44
SB-1B	101.29	6029.73	102.46	2728.7	88.55	4259.98
SB-1C	101.16	3906.44	102.64	1217.46	88.5	4286.37
SB-1D	101.41	3879.85	102.7	3873	88.67	3633.52
SB-1X	100.92	3844.01	102.49	294.01	88.27	4182.84
SB-1Z	101.19	6149.79	102.49	2923.11	88.43	5162.52
SB-1J	101	3930.5	102.42	904.3	88.32	3975.64
SB-1K	101.21	6792.9	102.6	2142.8	88.52	6670.03
SB-2A	101.1	853.6	102.37	281.73	88.5	1094.75
SB-2B	101.61	2260.66	102.87	7829.97	88.67	847.84
SB-2C	101.08	1221.22	102.62	159.87	88.4	1059.14
SB-2D	101.34	2861	102.72	4952.9	88.57	2244.2
SB-2X	101.11	1008.9	102.66	171.74	88.38	864.3
SB-2Z	101.43	3884.8	102.76	5733.9	88.65	2425.21
SB-2J	101.07	2039.34	102.51	395.39	88.39	1927.73
SB-2K	101.49	3151.5	102.71	5495.1	88.67	2096.51
